# Cognitive Flexibility in Schoolchild Through the Graphic Representation of Movement

**DOI:** 10.3389/fpsyg.2020.624922

**Published:** 2021-01-13

**Authors:** MᵃLuz Urraca-Martínez, Sylvia Sastre-Riba

**Affiliations:** Department of Educational Sciences, University of La Rioja, Logroño, Spain

**Keywords:** Neuroconstructivism, mental representation, drawing, schoolchild, education, movement, flexibility

## Abstract

Neuroconstructivism postulates the progressive complexity of mental representation over the course of cognitive development and the role of the graphic representation of movement in the transformation of mental schemas, cognitive flexibility, and representational complexity. This study aims to: (1) understand children’s resources in the drawing of movement (5–8 years); and (2) verify whether there are differences in the graphic representation of movement as an indicator of cognitive flexibility. The participants were *N* = 240 children aged 5–8 years; 1,440 drawings were collected representing 2,880 characters (both animate and inanimate) from six stories. The analysis consisted: (1) data quality control, using the kappa coefficient, and Generalizability Theory to test the instrument’s validity and reliability; (2) Multivariate General Analysis and Mixed Linear Analysis of the factors (age and stories); (3) Multivariate General Analysis of the graphic components: categories and microcategories, as well as the elements that make up the macrocategories: “Static,” “Indication,” and “Movement”; and (4) calculation of the generalizability coefficient (G-coefficient). The results show that: (a) age best explains variability, with a high effect size (η*^2^* = 0.732) across all components (*F* = 153.445; *p* < 0.001), thus increasing its complexity and (b) at ages 6 and 7, “Indication” appears as a modulator of “Static” (age 5) toward “Movement” (age 8). The generalizability coefficient is optimal (0.995). It is concluded that changes in the initial graphic representation of movement may interactively transform mental representation, thus increasing cognitive flexibility and prompting teaching applications to optimize such changes.

## Introduction

From a neuroconstructivist approach (Johnson, 2011; Dumontheil and Mareschal, 2020), cognitive development emerges as a dynamic and contextualized change in neural structures that enable the emergence of increasingly complex mental representations, supported by multiple brain regions and at different temporal scales, in response to the person’s proactive interaction with their physical and social environment (probabilistic epigenesis). This successive transformation follows a trajectory that begins with the initial constraints of neural structures, giving rise to different developmental stages throughout life (Sastre-Riba, 2014).

In this differential trajectory, mental representation is a key element for cognitive development and for understanding the emergence of child drawing, and changes thereof, as a graphic representation of internalized models of reality (Sirois et al., 2008). Child drawing is therefore the first marker that enables the study of mental representation as an external manifestation of internalized reality, by showing what is known about it.

It has been shown that events are naturally more attractive than objects, and their foremost feature is their movement. Therefore, part of the content of the first mental representations turns around the identity of events, objects, and people, and their movement and position, which forms the basis of the dynamic representations produced. The first external representative manifestation is the child’s scribble, in which the action of drawing already contains expressive and representational meanings relating to shapes, movements, and emotions (Quaglia et al., 2015), even if there is no real figure that relates to meaningful movement for representational purposes. Current studies are in agreement in supporting this early emergence (Matthews, 2010; Panesi and Morra, 2018).

Research on rigidity and flexibility in drawings (premanipulation) (Picard and Vinter, 2007) and their possible inhibitory or facilitating role in representational change (Barlow et al., 2003) remains scarce. Some authors argue that young children may have access to their own drawing procedures and show some flexibility in them (Lange-Küttner and Friederici, 2000; Burkitt and Barrett, 2010), inserting new elements earlier than proposed by Karmiloff-Smith’s (1999) representational redescription (RR) model, which suggests that procedural rigidity may be associated with the permanent notational trace or subroutines in drawing.

The three resources that may explain an increase in the development of graphic flexibility are (Cox, 2013; Allen et al., 2016): the availability of external models, endogenous changes in mental representations, and the theory of graphic representation among non-expert children (Berti and Freeman, 1997). The second of these would be comparable to RR, although it does not imply that early representations are necessarily rigid procedures, but that they may be transformed flexibly.

As an alternative to the RR model, some studies propose (Adi-Japha et al., 2010) the adoption of an integrative form that considers changes in information processing regarding control, executive functioning, and task complexity, along with the interaction between all of these, and their influence throughout development. Specifically, Morra (2005) puts forward three influencing factors: (a) the amount of attentional resources (M capacity) that a child can use to activate operative and figurative schemas in the relevant tasks; (b) the automatic activation of figurative schemas from perceptual input; and (c) executive activation, which sets appropriate objectives and monitors performance, emphasizing the relationship of working memory with children’s capacity to plan drawings and with their skill to modify usual schemas therein. Authors like Diamond et al. (2007) propose the special role of cognitive flexibility (also called shifting) as one of the core elements of executive functions because it is relate to mental flexibility, mental set shifting and closely linked to creativity. Other authors, in a similar sense, put forward factors associated with other executive functioning elements such as planning, monitoring, inhibition, and working memory (Riggs et al., 2013; Morra and Panesi, 2017; Panesi and Morra, 2020) thereby supporting previous hypotheses (Cox et al., 2001).

In addition, Braswell and Rosengren (2008) examine the role of biomechanical, cognitive, and contextual constraints in the development of drawing in order to understand how it may be conditioned by certain constraints.

As such, drawing is a complex representative skill that involves biomechanical, graphomotor, perceptive, cognitive, and social skills (Braswell and Rosengren, 2008; Salsa and Vivaldi, 2017). It is the outcome of multiple factors, such as underlying representational processes (Emmorey et al., 2008), attention to detail (Lange-Küttner et al., 2002), and executive functioning and its components, including working memory (Morra and Panesi, 2017), inhibition (Riggs et al., 2013), and flexibility.

In this vein, other authors have highlighted the lack of a thorough quantification of the graphic signs of movement typical of each stage of child development (Picard and Vinter, 2007) and of the levels of rigidity in the graphic representation of movement in usual drawings (premanipulation). On the other hand, Hollis and Low (2005) consider that most studies involve tasks that children cannot easily relate to, and which predetermine a lack of understanding or engagement in carrying them out.

In short, as it is a very important issue for understanding representational transformation, the lack of studies on the graphic representation of movement demands (Spelke, 2005; Mandler, 2008; de Hevia et al., 2014) further research in order to understand its genesis and progress, during a period that may lie between the ages of 5 and 8 years. This would enable an understanding of graphic representational processes as indicators of changes in cognitive development. In other words, capturing the signs of movement as part of a continuum of changes and transformations would allow us to infer the underlying representational restructuring process in children’s minds.

In accordance with the above, the aim of this study is two-fold: (1) to understand the differences in resources during the development (5–8 years) of the drawing of movement according to Age, Story, and their interaction and (2) to verify whether there are differences in the representation of movement as an indicator of cognitive flexibility. The main hypothesis is that there would be changes in the representation of movement in children’s drawing from the age of 5 influenced by cognitive flexibility, which would promote changes in internal representations.

## Materials and Methods

### Participants

The sample, extracted using intentional non-probabilistic sampling, comprised *N* = 240 schoolchildren aged 5–8 years with typical development, and was balanced with *n* = 60 participants per age group. A total of 1,440 drawings were collected, featuring a total of 2,880 characters. No participant received financial compensation of any kind.

### Materials

The stimulus material, adapted from Munuera (1999), consisted of six stories explicitly presenting action scenes that children could relate to, and which required movement to be graphically represented with varying degrees of difficulty, depending on whether or not the characters in each story have their own movement. Specifically, the content could be: (a) animate, i.e., stories in which both characters moved of their own accord or (b) mixed, where one of the characters was animate and the other was inanimate and had to be made to move. The names of the stories were as follows: (1) Two Rabbits, (2) Rabbit and Butterfly, (3) Rabbit and Wolf, (4) Two Persons, (5) Person and Bus, and (6) Person and Ball.

These stories met the following requirements: (1) evoking a complex movement, consisting of two simultaneous actions and (2) graphically representing movement, claiming the child’s involvement in the story.

An *ad hoc* coding instrument to capture movement in the drawings was adapted and validated according the Systematic Observational Methodology paramaters (Urraca-Martínez, 2015). This consisted of a mixed system of field formats and categories (Anguera et al., 2001), made up of the following components: (a) *n* = 3 macrocategories: “Static,”” Indication,” and “Movement”; (b) *n* = 12 categories comprising different positions-orientations; and (c) *n* = 49 microcategories as corporal and external indicators.

The categories consisted of the positions-orientations: Vertical front (Vf), Vertical back (Vb), Vertical front face profile (Vffp), Vertical full profile (Vfp), Horizontal front (Hf), Horizontal back (Hb), Horizontal front face profile (Hffp), Horizontal full profile (Hfp), Front tilt (Ft), Back tilt (Bt), Front face profile tilt (Ffpt), and Full profile tilt (Fpt). The microcategories comprised two types of indicators: Corporal (C) (e.g., articulated arm, stretched leg, etc.) and External (E) (e.g., scrollworks, lines, etc.).

The microcategories, combined with the above-described categories, enabled the graphic representation of participants’ drawings to be encoded as follows: “Static,” when there was no indicator of movement; “Indication,” when a precursor indicator of the expression of movement appeared; and “Movement,” when movement was clearly expressed in the figures drawn. On the other hand, since the stories featured two characters, the following modalities arose, depending on the combination of macrocategories defining the type of movement represented by each character: Static/Static, Static/Indication, Static/Movement, Indication/Indication, Indication/Movement, and Movement/Movement. The categories that conform the instrument are nested exhaustively for each one of the criteres that conform the mixed system of field formats and categories.

Indication refers to the introduction in children’s drawings of some lines, spirals, etc., expressing no rigidity in the static figure, as a precursor indicator of the expression of movement. Cognitive flexibility is measured according to the specialized literature through the specific microcategories of the mixed system of analysis. The evaluation of the appearance of indicators is the result of the application of this mixed analysis system, allowing their empirical concretion and differential operationalization based on the interobserver realibility. All of the above made it possible to rigorously capture the level of representation of movement.

### Measure and Data Analyses

The study was administered to each school group (years 2 and 3 of early childhood education; years 1, 2, and 3 of primary education, in order to ensure the presence of all ages range), during school hours. The administration interval ranged from 60 to 75 min. As a prompt, the researcher told each of the stories, stressing the aspects involving movement and asking participants, at the end of each story, to draw it.

The data analysis plan consisted of:

1. Data quality control. *n* = 28 drawings were randomly drawn from each of the four groups of participants, based on the age being studied, and from each of the six types of story administered. The Aleatori 1.0 program (Vargas, 1999) was used.

Cohen’s kappa coefficient (Cohen, 1960) was calculated to measure inter-rater reliability. In order to calculate the validity of the coding instrument, the G-coefficient was identified using a two-faceted measurement plan: Observers^*x*^Categories. The Ysewijn’s (1996) Generalizability Theory (GT) program was used.

• Analysis of the factors Age and Stories by means of: (a) the Multivariate General Linear Model (GLM) to verify the interaction, significance, and the effect size and (b) the Mixed Linear Model (MLM) to estimate the facets of variability.
2. Multivariate analysis of the components of categories, microcategories, and modalities.
3. Calculation of the effect size for the components “Static,” “Indication,” and *“*Movement” of the macrocategories by the Multivariate Lineal General Analysis. The SPSS Statistics 24.0 program was used for analyses 2, 3, and 4.
4. Calculation of the generalizability of the results using the G-coefficient (Cronbach et al., 1972), with a measurement plan in which participants constituted the instrumentation or generalization facet, while Age and Stories composed the differentiation facet. The Ysewijn’s (1996) GT program was used.

## Results

Regarding data quality control, Cohen’s kappa coefficient was *k* = 0.802. The generalizability study revealed that the reliability of the results was optimal (0.999) with values close to 1, and the value of the interaction of the two facets Category^*x*^Observer (C*^*x*^*O) was 0%, thus accounting for most of the variance.

The validity of the coding instrument reported a generalizability coefficient of (0.000), with a variability of 100% for the Category facet and null for the Observer facet and for the interaction between the two, so a highly significant category goodness was estimated. This confirmed the instrument’s consistency. Table 1 shows these values.

**TABLE 1:** Interrater reliability and validity of the coding instrument.

	***F***	***SS***	***VC***	**%**	***CG***
Reliability	O	0.35	0.00490	0	0.999
	C	4962.96	48.61086	100	
	CO	4.65	0.09125	0	
	***V***	***SC***	***CV***	**%**	***CG***
	O	0.04	0.00000	0	0.000
Validity	C	5019.65	49.19306	100	
	OC	1.96	0.03846	0	

*O, Observer; C, Category; SS, Sum of squares; VC, Variance components; GC, Generalizability Coefficient.*

Table 2 shows the initial approximation of the values of the effect size of Age and Stories in the representation of movement, indicating that: (a) Age influences changes in the representation of movement in child drawing [*F*_3_ = 214.695; *p* ≤ 0.001] with a high effect size (η^2^ = 0.732); (b) for Stories, the values of the effects between them are statistically significant [*F*_4_,_010_ = 28.052; *p* ≤ 0.001; η^2^ = 0.106], therefore the representation of movement differs according to its content; and (c) there are statistically significant changes [*F*_12_,_029_ = 4.932; *p* ≤ 0.001; η^2^ = 0.059] in the Age^*x*^Stories intersection with a low effect size.

**TABLE 2:** Multivariate linear analysis: age and stories.

**Factors**	***F***	***DF***	***p***	**η *^2^***
Age	214.695	3	<0.001	0.732
Stories	28.052	4.010	<0.001	0.106
Age^*x*^Stories	4.942	12.029	<0.001	0.059

These results indicate that: (a) the greatest effect is that of Age, rather than the content of the Stories, with a smaller magnitude in the interaction between the two; (b) changes in drawing that involve expressing more indicators of movement with age demonstrate flexibility; and (c) the stories include content that facilitates, to a greater or lesser extent, the graphic representation of movement across all ages.

Table 3 shows the results of the analysis (MLM), providing a more precise view of the influence of Age and Stories on the representation of movement. Specifically, taking into account the four ages under study and the six stories, the contrast of fixed effects is statistically significant for Age (*F* = 153.445; *p* < 0.001) and for Stories (*F* = 10.366; *p* < 0.001), but not for their intersection (*F* = 0.798; *p* = 0.681). In other words, both Age and the content of Stories influence changes in the representation of movement, corroborating the previous findings that there are contents that, at any ages, encourage a greater or lesser extent the representation of movement.

**TABLE 3:** Mixed multivariate linear analysis of age and stories.

	***F***	***p***	**σ ^2^**	***Wald Z***	***P***
A	153.445	<0.001			
St	10.366	<0.001			
A*St	0.798	0.681			
R			4.140	26.777	<0.001
			1.729	26.796	<0.001

*A, Age; St, Stories; R, Residual.*

On the other hand, a Mixed Multivariate Lineal Analysis of the estimation of the effect of Age confirms that the representation of movement increases with age (γ*_5_* = −01.133, γ*_6_* = −0.716; γ*_7_* = 0.400; γ*_8_* = 0.011) (*p* < 0.001) and, after controlling for the Stories factor, the representation of movement differs with Age by 95%. However, after controlling for the Age factor, the content of Stories only produces an effect of 6%. In other words, the effect of Age has the most influence on how movement is graphically represented.

As for the components, statistically significant values are obtained for all of these across all Stories with intervals between (*F* = 2.61; *p* < 0.001) and (*F* = 11.14; *p* < 0.001). This result demonstrates their influence on changes in the representation of movement in child drawing. The values in global scores point to the influence of the positions and orientations (*F* = 27.43; *p* < 0.001); corporal indicators (*F* = 10.70; *p* < 0.001) and modalities (*F* = 32.64; *p* < 0.001) as resources that children use to graphically represent movement, either statically or dynamically, in their drawings.

Finally, Table 4 shows the results relating to the macrocategories (“Static,” “Indication,” and “Movement”) and the influence of the Age^*x*^Stories intersection to graphically signal movement. The results indicate that both Age (*F*_*s=*_217.416; *p* < 0.001;η^2^ = 0.315; *F_*i*_* = 9.278; *p* < 0.001; η^2^ = 0.019 and *F_*m*_* = 163.464; *p* < 0.001); η^2^ = 0.257 and Stories (*F_*e*_* = 11.339; *p* < 0.001; η^2^ = 0.038; *F_*i*_* = 7.577; *p* < 0.001 η^2^ = 0.026 and *F_*m*_* = 10.231; *p* < 0.001); η^2^ = 0.035 influence changes in the drawing of the “Static,” “Indication,” and *“*Movement,” but this is not the case for the Age^*x*^Stories intersection (*F_*s*_* = 0.994; *p* = 0.458; η^2^ = 0.010; *F_*i*_* = 1.462; *p* = 0.111; η^2^ = 0.015 and *F_*m*_* = 0.768; *p* = 0.692); η^2^ = 0.008.

**TABLE 4:** Multivariate linear analysis of macrocategories: static, indication, and movement.

**Factors**	**Macrocategories**	***F***	***p***	***η ^2^***
Age	Sta	217.416	< 0.001	0.315
	Ind	9.278	< 0.001	0.019
	Mov	163.464	< 0.001	0.257
Stories	Sta	11.339	< 0.001	0.038
	Ind	7.577	< 0.001	0.026
	Mov	10.231	< 0.001	0.035
Age*^*x*^*Stories	Sta	0.994	0.458	0.010
	Ind	1.462	0.111	0.015
	Mov	0.768	0.692	0.008

*Sta, Static; Ind, Indication; Mov, Movement*

These results show, yet again, that both age and the content of stories influence the use of “Indication,” “Static,” and “Movement” in drawing, but this is not the case for their intersection. The results that follow further establish this. Age is what best accounts for the variability of the results found (to the tune of 58%). Within this, the “Static” macrocategory accounts for up to 31% of said variability, that of “Movement” accounts for 26%, and that of “Indication” for just 2%.

**FIGURE 1:**
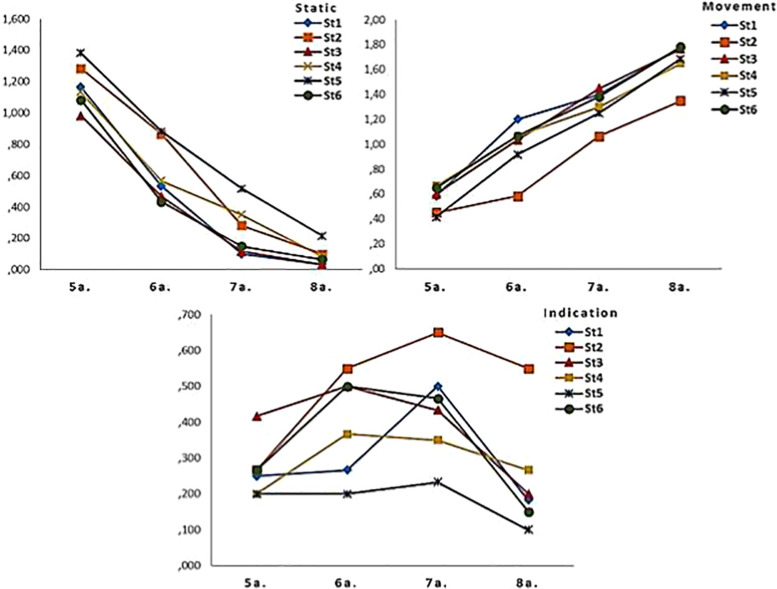
Representation of macrocategories according to age.

All of this is presented in Figure 1, which shows a linear trend for age in the drawing of movement, which is predominantly characterized by the “Static” macrocategory at age 5, and by that of “Movement” at age 8, with a noticeable decline in “Static” as age increases, while “Indication” increases at ages 6 and 7 and declines at age 8. In specific terms, *“*Static” representation is dominant at age 5, while the increase in “Indication” at ages 6 and 7 suggests a phase of transition toward “Movement,” which is dominant at age 8. Therefore, not only does the dynamic representation of movement increase progressively with age, but children show flexibility by redefining their drawing strategy.

**FIGURE 2:**
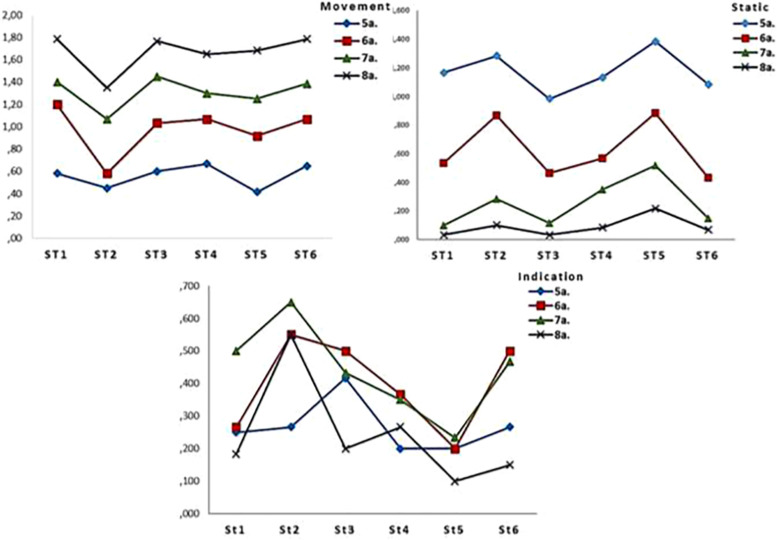
Representation of macrocategories according to stories.

As regards the role of stories and their content, Figure 2 shows a non-linear trend. While there are differences in representation as “Static” and as “Movement” for each story between the different ages, this follows a common trend: there are more drawings that make use of the “Static” indicator for story 5, but “Movement” is dominant for story 3 (except at age 5); “Indication” is dominant for story 1 (except at age 5), but is lower for story 2 at all ages. This explains why there are no significant differences in the Age^*x*^Stories (A*^*x*^*S) factor.

As we have seen, “Indication” appears significantly at ages 6 and 7, as an indicator of the transition in children’s skills in graphically representing movement, from “Static” at age 5 toward “Movement” proper at age 8.

The generalizability of these results is guaranteed given the high generalizability coefficient obtained (0.995), marginally improved upon in the optimization plan, which considers *n* = 80 and 100 participants for each age group studied.

## Discussion

This study highlights changes in the representation of movement in child drawing from age 5–8, indicating the early flexibility and graphic resources of children at age 5, confirming the hypotheses proposed. Age-related differences related could be the result of the construction of new representations based on existing ones, thus explaining the continuous process of mental transformation (Sirois et al., 2008; Mareschal and Westermann, 2010). These progressive changes (Cox et al., 2001; Picard and Vinter, 2007) show that flexibility in child drawing increases with age, in line with the greater structural and functional complexity of cognitive development.

The results agree with those of other studies into the role of cognitive flexibility in the representational changes expressed in child drawing throughout development, based both on the RR approach (Karmiloff-Smith, 1999) and on that of endogenous changes in mental representations (Berti and Freeman, 1997), recursive re-representation (Spensley and Taylor, 1999), or the effect of executive functions (Miyake et al., 2000; Jolley et al., 2013; Morra and Panesi, 2017).

At the same time, the results are in line with authors who suggest the existence of cognitive flexibility from ages 5 or 6 (Freeman and Adi-Japha, 2008), noting that, from age 5, children are able to reorder their subroutines within process restriction (Barlow et al., 2003; Allen et al., 2016) and to modify their usual graphic schemas, showing flexibility in producing their drawings (Adi-Japha et al., 2010) with the aim of graphically representing the movement of the key characters and elements involved in a story (Cox, 2005; Quaglia et al., 2015). Results obtained show that since age 5 there are indicators thereof in their attempt to represent “Movement” in drawing using “Indication,” which points to their inhibition of “Static” rigidity. Moreover, changes in drawing at all ages studied reveal the emergence of increasingly elaborate resources (as highlighted by “Indication”), which play a role in both external and internal representation in their attempts at graphic expression in images, until they are successfully able to draw clear signs of movement in said images, according to Braswell and Rosengren, 2008; Mareschal and Westermann, 2010). This supports the hypotheses of the early emergence of cognitive flexibility (Bialystok et al., 2006) and the capacity to adapt to new demands through the other executive components of inhibition and working memory (Diamond et al., 2007), in keeping with a neuroconstructivist approach (Westermann et al., 2007; Sirois et al., 2008; Dumontheil and Mareschal, 2020).

Although there are different ways to represent movement at different ages and for different stories, the tendency is to use these components according to the content, across all ages. Moreover, in support of other studies (Hollis and Low, 2005; Cox, 2013), story content is shown to have an influence across all ages as an extrinsic motivator in the graphic representation of movement, in particular increasing the number of indicators used when there are two animate characters and when the topic is one that children can relate to.

As a contribution of this study, it is worth stressing the existence of “Indication” as a graphic indicator situated on the continuum between “Static” drawing (at age 5) and drawing showing “Movement” proper (at age 8). “Indication” has high prevalence at ages 6 and 7, but declines thereafter, practically disappearing at age 8, when the child is now equipped with other procedures to graphically represent the movement of characters and objects. At the same time, this is an important factor that supports the argument of child flexibility (Bialystok et al., 2006; Freeman and Adi-Japha, 2008; Matthews, 2010; Allen et al., 2016).

This contribution may also relate to studies that indicate that children at age 5 are metacognitively satisfied with the representation of movement they achieve (Bonoti and Metallidou, 2010), without adding changes to what they have drawn, despite limitations in the dynamic expression achieved, but that, starting at age 6, given greater awareness of the task’s requirements and greater regulation of their resources, they attempt to modify the available schema in order to solve the task in question (Touroutoglou and Efklides, 2010). “Indication” is therefore a resource that enables the graphic representation of movement when the child, despite having a mental representation of the movement, does not possess sufficient graphic strategies to modify the characteristics of the figure in order to convey it.

As a conclusion, graphically representing movement may prompt a change in the representation of reality (Braswell and Rosengren, 2008; Rose and Jolley, 2020), since its external reconstruction may interactively transform the mental representation, thereby increasing cognitive flexibility (Freeman, 2004; Cox, 2013). If the manifestation of external representations expresses the transformation of internal representations through the progressive increase in the capacities of the cortex in interaction with external events (Karmiloff-Smith, 2009), we can see the relevance of adjustments in the graphic representation of movement for progress in the management of internal representations, as well as the role of partial representations that facilitate more complex changes, which are determined by proactivity and progressive specialization (Sirois et al., 2008).

This suggests some educational applications aimed at optimizing changes in the graphic representation of movement between ages 5 and 6, which may functionally encourage the redefinition of internal representation, that is, cognitive change relating to knowledge of reality and its organization.

## Data Availability Statement

The raw data supporting the conclusions of this article would be made available by the authors.

## Ethics Statement

The studies involving human participants were reviewed and approved by University of La Rioja. Written informed consent to participate in this study was provided by the participants’ legal guardian/next of kin.

## Author Contributions

SS-R supervised the study, analyzed the data, and wrote the manuscript. M-U-M collected the data, performed the analyses, and contributed to the writing of the manuscript. All authors revised and approved the submitted version.

## Conflict of Interest

The authors declare that the research was conducted in the absence of any commercial or financial relationships that could be construed as a potential conflict of interest.
